# Evaluation of Reference Genes for Real-Time Quantitative PCR Analysis in Larvae of *Spodoptera litura* Exposed to Azadirachtin Stress Conditions

**DOI:** 10.3389/fphys.2018.00372

**Published:** 2018-04-11

**Authors:** Benshui Shu, Jingjing Zhang, Gaofeng Cui, Ranran Sun, Veeran Sethuraman, Xin Yi, Guohua Zhong

**Affiliations:** Key Laboratory of Crop Integrated Pest Management in South China, Ministry of Agriculture, Key Laboratory of Natural Pesticide and Chemical Biology, Ministry of Education, South China Agricultural University, Guangzhou, China

**Keywords:** *Spodoptera litura*, azadirachtin, RT-qPCR, reference gene, stability

## Abstract

Azadirachtin is an efficient and broad-spectrum botanical insecticide against more than 150 kinds of agricultural pests with the effects of mortality, antifeedant and growth regulation. Real-time quantitative polymerase chain reaction (RT-qPCR) could be one of the powerful tools to analyze the gene expression level and investigate the mechanism of azadirachtin at transcriptional level, however, the ideal reference genes are needed to normalize the expression profiling of target genes. In this present study, the fragments of eight candidate reference genes were cloned and identified from the pest *Spodoptera litura*. In addition, the expression stability of these genes in different samples from larvae of control and azadirachtin treatments were evaluated by the computational methods of NormFinder, BestKeeper, Delta CT, geNorm, and RefFinder. According to our results, two of the reference genes should be the optimal number for RT-qPCR analysis. Furthermore, the best reference genes for different samples were showed as followed: EF-1α and EF2 for cuticle, β-Tubulin and RPL7A for fat body, EF2 and Actin for midgut, EF2 and RPL13A for larva and RPL13A and RPL7A for all the samples. Our results established a reliable normalization for RT-qPCR experiments in *S. litura* and ensure the data more accurate for the mechanism analysis of azadirachtin.

## Introduction

Real-time quantitative polymerase chain reaction (RT-qPCR) is considered to be the reliable and effective method for the quantitative analysis of candidate genes expression level and the verification of transcriptomic analysis, especially in species which lacking the genomic information, due to the advantages of high sensitivity and specificity, more convenience and good reproducibility (Ibanez and Tamborindeguy, 2016; Zhang et al., 2017). However, some factors including RNA integrity and quality, reverse transcription efficiency and primer amplification efficiency could interfere and influence the accuracy and reliability of RT-qPCR. Therefore, the reference genes attracted attention and were used for the precise normalization (Zhang et al., 2015; Arya et al., 2017). The idealized reference genes which defined as the “constitutively expressed to maintain cellular function” should have the relatively stable expression under various tissues and physiological conditions (Pan et al., 2015; Chen et al., 2016). Simultaneously, the expression levels of reference genes might vary under different experimental conditions. Selection of appropriate reference genes is the prerequisite to ensure the accuracy of experimental results (Nagy et al., 2017). Of course, standardizing experimental results with two or more reference genes could also improve the accuracy and be recommended (Shi et al., 2016).

Previous studies have shown that the basic metabolism genes including actin, tubulin, ribosomal protein, glyceraldehydes 3-phosphate dehydrogenase, elongation factor have been used as the reference genes for RT-qPCR (Płachetka-Bozek and Augustyniak, 2017; Wan et al., 2017). However, recent reports indicated that reference genes also have independent functions and were involved in various physiological and pathological processes, such as GAPDH and 18S rRNA (Nicholls et al., 2012; Kozera and Rapacz, 2013). Azadirachtin, a proverbial tetranortriterpenoid, was considered to be the most promising botanical pesticide for pest control with the effect of antifeedant, insect growth and development inhibition (Shu et al., 2015; Wang et al., 2015). It was demonstrated that some basic metabolism genes were affected by azadirachtin. In *Drosophila melanogaster*, immunohistochemistry and *in silico* analysis confirmed that azadrachtin bind to actin and inhibited its polymerization, which indicated that actin could be act as the putative target of azadirachtin (Anuradha et al., 2007; Pravin Kumar et al., 2007). Simultaneously, azadrachtin bind to actin was verified in *Plutella* (Anuradha and Annadurai, 2008). Further report showed that the cytoskeletal function was also influenced by azadirachtin (Huang et al., 2010).

As the most serious polyphagous insect pest, *Spodoptera litura* has the characteristics of high fertility, short life cycle, abundant host plants, devastating for many economic crops and widely distributed in the tropical and subtropical Asia (Bano and Muqarab, 2017; Feng et al., 2017; Kaur et al., 2017). Besides, the problems of pest resistance produced by frequent and irrational use of chemical insecticides are also more prominent (Sang et al., 2016; Lin et al., 2017). Azadirachtin has the significant antifeedant and growth inhibitory action against *S. litura* (Jeyasankar et al., 2011). Recently, the discovery and publication of *S. litura* genome data have provided new insights into many biological problems including the mechanisms of evolution and resistance, the specialization of host plants and ecological adaptation (Cheng et al., 2017). It also renewed interests in interpreting the mechanisms of azadirachtin against *S. litura* at the molecular level.

In this study, in order to verify the suitable reference genes of *S. litura* for RT-qPCR under azadirachtin treatments, eight candidate reference genes actin (Actin), elongation factor 1alpha (EF-1α), elongation factor 2 (EF2), glyceraldehyde 3-phosphate dehydrogenase (GAPDH), ribosomal protein L7A (RPL7A), ribosomal protein L13A (RPL13A), alpha-tubulin (α-tubulin), beta-tubulin (β-tubulin) were identified and cloned from *S. litura*. The expression stabilities of eight reference genes in larva, cuticle, fat body, and midgut samples from larvae of control and different concentrations of azadirachtin treatments were measured by five programs (NormFinder, BestKeeper, Delta Ct method, geNorm, and RefFinder). This study could potentially reveal the expression variations of reference genes in response to azadirachtin, which could provide some foundations for RT-qPCR analysis of *S. litura* in the future.

## Materials and methods

### Insects

The third-instar larvae of *S. litura* fed with a standard artificial diet were used as control group and the azadirachtin-treatment group were the third-instar larvae fed with the diet added azadirachtin (1.0, 2.5, and 5.0 mg/g) for 7 d. All the larvae were kept in the conditions of 25 ± 1°C, 60–70% relative humidity and a 16:8 h light: dark cycle in Key Laboratory of Natural Pesticide and Chemical Biology, Ministry of Education, South China Agricultural University.

### Sample collection

After feeding with artificial diet for 7 d, some of the larvae with different treatments (*n* = 5) were collected and kept in −80°C and some were dissected (*n* = 20). The cuticle, fat body and midgut were separated and washed in cold phosphate buffered saline (PBS), then collected and kept in −80°C. For the tissues or larva samples, three biological replications of each treatment were collected.

### Total RNA isolation and cDNA synthesis

All the samples were ground into powder by liquid nitrogen and 1 ml RNAiso plus (Takara, Japan) was added for total RNA isolation following the experimental procedures. Samples were mixed well and lysed for 5 min at room temperature, 200 μL chloroform was added and shaken for 15 s. The mixture was incubated for 3 min at room temperature. After centrifuged with 12,000 rpm for 10 min at 4°C, 500 μL supernatant was separated and mixed with 500 μL isopropanol, incubated for 10 min at room temperature, then centrifuged with 12,000 rpm for 10 min at 4°C. Removed the supernatant and 75% ethanol was added and washed the precipitate. After centrifuged, the ethanol was removed and the precipitate was dried at room temperature for 3 min, an appropriate amount of DEPC water was added to dissolve the precipitate. The concentration and purity of total RNA were measured by a NanoDrop® spectrophotometer (Thermo Fisher, MA, USA).

Qualified RNA (1 μg) was used for cDNA synthesis by PrimeScript RT reagent Kit with gDNA Eraser (TaKaRa, Japan) following the manufacturer's instructions. A total 10 μL reaction system contained 1 μg total RNA, 2 μL 5 × gDNA Eraser Buffer and 1 μL gDNA Eraser was incubated for 2 min at 42°C, and then kept at 4°C. Another 10 μL reaction solution were prepared with 4 μL RNase free water, 4 μL 5 × PrimeScript Buffer 2, 1 μL RT Primer Mix and 1 μL PrimeScript RT Enzyme Mix I and then mixed with the solution as above, the admixture was incubated at 42°C for 15 min, 85°C for 5 s and stored at −20°C.

### RT-PCR and RT-qPCR analysis

According to the transcriptome of *S. litura* (GenBank number: GBBY00000000) (Gong et al., 2015), eight genes (Actin, EF-1α, EF2, GAPDH, RPL7A, RPL13A, α-tubulin, β-tubulin) were selected as candidate reference genes. The RT-qPCR primers of those genes were designed by Primer Premier 5.0 (Premier, Canada). The primers were used to clone the fragments with LA *Taq* (Takara, Japan). The amplification was fulfilled by denaturing at 95°C for 3 min, followed with 32 cycles of 95°C for 30 s, 60°C for 15 s, and a 10 min extension at 72°C. The PCR products were checked by 1.5% agarose gel electrophoresis. PCR products were ligated with pMD-19T and transformed into *Escherichia coli* DH5α. Plasmids were extracted by TIANprep Mini Plasmid kit (TIANGEN, China) and used as the templates for standard curve of reference genes. RT-qPCR was performed by iTaq^TM^ Universal SYBR® Green Supermix (BIO-RAD, USA) in the CFX Connect^TM^ Real-Time System (BIO-RAD, USA). The reaction procedure was consists of the following steps: a denaturation step at 95°C for 3 min, followed with 95°C 10 s; 60°C 10 s; 72°C 15 s for 40 cycles, and ended with a melting-curve analysis. For the PCR efficiency, a series of 10-fold serially diluted plasmids were used as templates and the procedure for RT-qPCR was performed as above.

### Data analysis

All the *Ct*-values from RT-qPCR were collected and the stability of reference genes were analyzed by the software of NormFinder, BestKeeper, Delta CT, geNorm, and RefFinder. NormFinder could identify the optimal normalization reference genes according to the direct measure to estimate expression variation and verify the expression stability (Andersen et al., 2004). BestKeeper is another algorithm for stability detection of reference genes and it could calculate by the amplification efficiency of primers and the *Ct*-values of reference genes (Pfaffl et al., 2004). The Delta ΔCT method evaluated the stability of reference genes by comparing the relative expression of pairwise genes within each sample (Silver et al., 2006). geNorm was used to evaluate the stability of candidate reference genes and determine the appropriate reference gene number in RT-qPCR. The lowest Ct value in the reference gene is very important for this algorithm and the stability value (M) calculated by geNorm was used to assess the stability of reference genes. Furthermore, the average geNorm *M* ≤ 0.2 was considered to be a reference for high reference genes stability. Besides, pair-wise variation value (V) calculated by geNorm was used for determine the optimal normalization factor number and geNorm *V* < 0.15 could be the standard for better normalization (Vandesompele et al., 2002). Furthermore, the powerful web-based analysis tool, RefFinder (http://www.leonxie.com/referencegene.php), was used to get the comprehensive ranking results for the stability assessment of reference genes by integrating the results of four software we used as above.

## Results

### Transcriptional profiling of candidate reference genes

**Figure 1:**
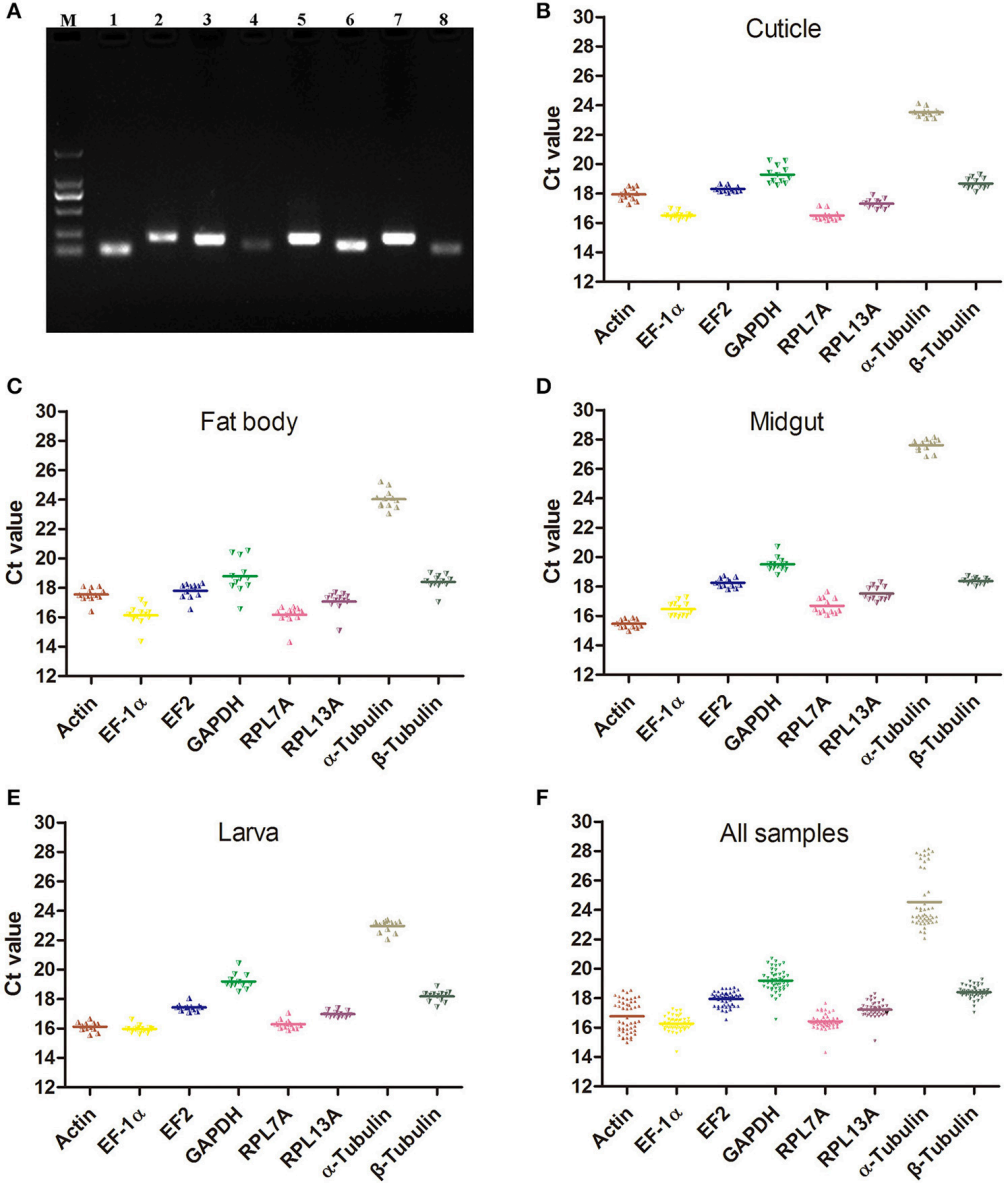
The expression profiles of the eight candidate reference genes. **(A)** Agarose gel of eight candidate reference genes PCR products amplified with the RT-qPCR primers. Lanes: M, Marker 2000, lane 1-8, Actin, EF-1α, EF2, GAPDH, RPL7A, RPL13A, α-Tubulin and β-Tubulin. **(B–F)** The expression profiles of the eight candidate reference genes in cuticle, fat body, midgut, larva, and all samples. (Actin), actin; (EF-1α), elongation factor 1alpha; (EF2), elongation factor 2; (GAPDH), glyceraldehyde 3-phosphate dehydrogenase; (RPL7A), ribosomal protein L7A; (RPL13A), ribosomal protein L13A; (α-Tubulin), alpha-tubulin; (β-Tubulin), beta-tubulin.

Before the expression stability evaluation, eight candidate reference genes belong to four functional groups including 3 of the structure-related genes (Actin, α-Tubulin and β-Tubulin), 2 of the ribosomal protein (RPL7A and RPL13A), 2 of the protein factor (EF-1α and EF2), and one metabolism-related gene (GAPDH) were cloned and identified by sequencing. All the PCR products amplified by the primers were detected with 1.5% agarose gel and the single band was observed in each PCR product with the expected strip size ranged from 106 to 222 bp (Figure 1A). All the gene primers were validated before the reference genes selected for normalization and the primer efficiency and correlation coefficient (*R*^2^) were calculated and showed in Table 1. The amplification efficiencies of primers met the standard requirement of conventional RT-qPCR, the primer efficiency was ranged from 92.4 to 101.1% and almost all the *R*^2^ of standard curve line were more than 0.995.

**Table 1:** Primer features of eight candidate reference genes for RT-qPCR in *S. litura*.

**Gene name (Abbreviation)**	**Accession No**.	**Primer Sequence**	**Product length (bp)**	**Tm (°C)**	**Primer efficiency (%)**	***R*^2^**	**Slope**
RpL13A		5′ TGTGGAAGACTGTCAGAGGCA 3′	106	60	97.2	0.998	−3.391
		5′ GGTTGTCATAGGGTGGTGGG 3′					
alpha-tubulin		5′ CAGATGCCCTCCGACAAGA 3′	113	60	92.4	0.999	−3.518
		5′ GGCTCCAAGTCCACGAACA 3′					
RpL7A		5′ CGCCCTTTGCCGTAAGAT 3′	222	60	95.3	0.997	−3.439
		5′ TTGTTGCCGAGGACACCAC 3′					
Actin	KP331524.1	5′ GTTGCTGCGTTGGTAGTAGACA 3′	202	60	95.5	0.999	−3.435
		5′ CGATGGGGTACTTGAGGGTAA 3′					
GAPDH	HQ012003.2	5′ TTGATGGACCCTCTGGAAAAC 3′	154	60	95.0	0.998	−3.448
		5′ TTAGCAACAGGAACACGGAAA 3′					
EF2		5′ ACCAAGAAGTGGGCGAAACA 3′	214	60	94.0	0.999	−3.473
		5′ GCAGCCAGCTACGCATAACC 3′					
EF1- alpha	KC007373.1	5′ CCCTGCCAACATCACCACT 3′	131	60	94.4	0.986	−3.464
		5′ CGTAACCACGACGCAACTCC 3′					
RpL10A		5′ CTCATCAAGCAGATCCCACG 3′	160	60	93.6	1.000	−3.486
		5′ CAGCCACAGACAGGCACAATA 3′					
18S ribosomal RNA	JX041463.1	5′ CCTGCTAAATAGGCGTCGTCA 3′	225	60	93.5	0.998	−3.488
		5′ AGGAGTTTCAGCGGGTTGC 3′					
beta-tubulin		5′ TTGCCTCCAAGGATTCCAAC 3′	135	60	101.1	0.995	−3.296
		5′ GGCGAAGGTACAACTGAGTATGTG 3′					

The raw *Ct*-values of eight candidate reference genes were detected by RT-qPCR and ranged from 14.31 (RPL7A) to 28.15 (α-Tubulin). The average *Ct*-values of Actin, EF-1α, EF2, GAPDH, RPL7A, RPL13A, α-Tubulin and β-Tubulin were 16.76 ± 1.08, 16.27 ± 0.50, 17.94 ± 0.49, 19.20 ± 0.76, 16.41 ± 0.50, 17.21 ± 0.49, 24.53 ± 1.89, and 18.40 ± 0.41, respectively (Figures 1B–F). All the threshold fluorescence peak of candidate reference genes were fall in the cycles of 15–30 and α-Tubulin has the lowest expression level.

### Identification of best reference gene for RT-qPCR

In this study, the stability of eight candidate reference genes was analyzed by five programs NormFinder, BestKeeper, Delta CT, geNorm, and RefFinder in different samples.

### Normfinder analysis

**Figure 2:**
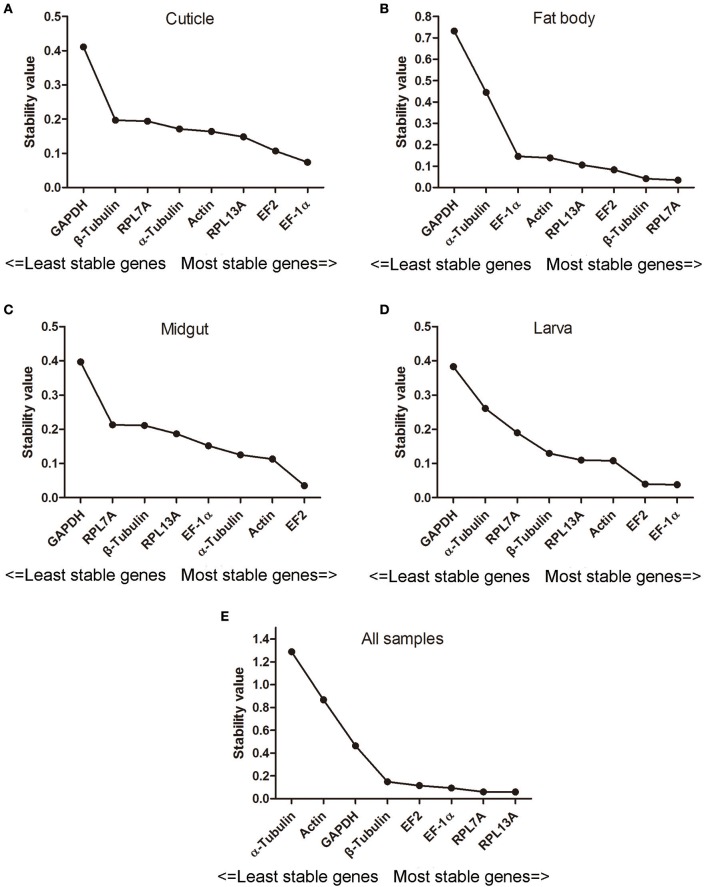
The calculation of eight candidate reference genes expression stability by NormFinder. Abbreviations were showed as in Figure 1. Panels **(A–E)** were represented as the stability validation results of reference genes expression in cuticle, fat body, midgut, larva, and all samples.

The stability of eight candidate reference genes evaluated by NormFinder software were depended on the stability value and the stability of reference genes was negatively correlated with the stability values. As shown in Figure 2, EF-1α, RPL7A, and EF2 were regarded as the most stable genes in the tissues of cuticle, fat body, and midgut, and the least steady genes in these tissues was GAPDH. In addition, EF-1α was the ideal reference gene and followed with EF2 and Actin for larva. For all the samples, the order of reference gene stability was: RPL13A > RPL7A > EF-1α > EF2 > β-Tubulin > GAPDH > Actin >α-Tubulin.

### BestKeeper analysis

BestKeeper determines the stability of reference genes based on the standard deviation (SD) value, the coefficient of variation (CV) and correlation coefficient (r). The reference gene with high stability has the lower SD value and CV, and higher correlation coefficient. Besides, the candidate gene with SD > 1 indicated unstable and could not be used as the reference. As shown in Table 2, the most and least stable reference genes in cuticle and larva was EF2 and GAPDH, respectively. Furthermore, Actin could be the most stable reference gene with the lowest SD value in the tissue of fat body and followed by β-Tubulin and EF2. In addition, the reference gene β-Tubulin was considered to be the most stable gene for the midgut samples. The reference gene stability arrangement in all samples was showed as followed: β-Tubulin > RPL13A > RPL7A > EF-1α > EF2 > GAPDH > Actin > α-Tubulin. These results indicated that single reference gene could not be the ideal reference for all organizations.

**Table 2:** Expression stability of the eight candidate reference genes in *S. litura* by BestKeeper algorithm.

**Gene name**	**Cuticle**			**Fat body**			**Midgut**			**Larva**			**All samples**		
	**Rank**	**Std dev [±CP]**	**Coff. of corr.[r]**	**Rank**	**Std dev [±CP]**	**Coff. of corr.[r]**	**Rank**	**Std dev [±CP]**	**Coff. of corr.[r]**	**Rank**	**Std dev [±CP]**	**Coff. of corr.[r]**	**Rank**	**Std dev [±CP]**	**Coff. of corr.[r]**
Actin	7	0.329	0.686	1	0.333	0.852	2	0.248	0.756	5	0.253	0.738	7	0.990	0.256
EF-1α	2	0.164	0.822	6	0.442	0.866	6	0.422	0.955	3	0.176	0.855	4	0.372	0.886
EF2	1	0.159	0.657	3	0.401	0.926	3	0.254	0.877	1	0.160	0.901	5	0.404	0.923
GAPDH	8	0.500	0.609	8	0.847	0.722	4	0.332	0.149	8	0.380	0.385	6	0.585	0.530
RPL7A	5	0.244	0.614	4	0.410	0.979	8	0.468	0.943	4	0.235	0.676	3	0.329	0.822
RPL13A	4	0.233	0.696	5	0.416	0.919	7	0.422	0.875	2	0.167	0.538	2	0.323	0.850
α-Tubulin	3	0.216	0.621	7	0.464	0.538	5	0.337	0.811	7	0.342	0.602	8	1.59	0.509
β-Tubulin	6	0.312	0.619	2	0.341	0.947	1	0.159	0.498	6	0.281	0.757	1	0.286	0.749

### Delta CT analysis

**Figure 3:**
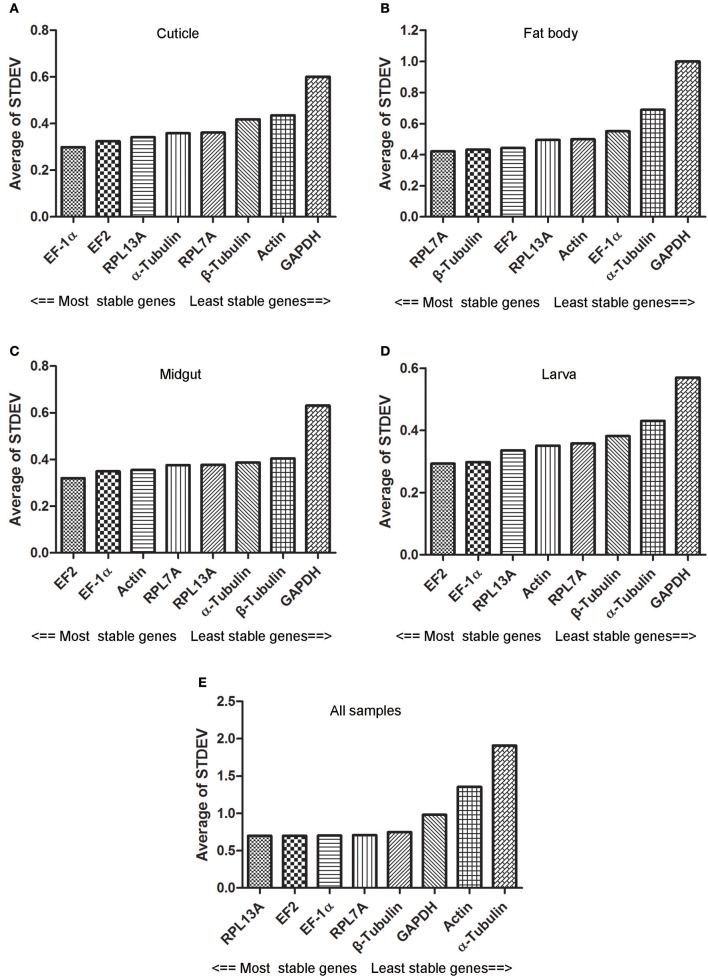
Expression stability of eight candidate reference genes evaluated by Delta CT method. Abbreviations were showed as in Figure 1. Panels **(A–E)** were represented as the stability validation results of reference genes expression in cuticle, fat body, midgut, larva, and all samples.

According to the results of Delta CT analysis, EF2 with the lowest STDEV was regarded as the most sable gene for the samples of midgut and larva. Simultaneously, the genes with the most stable expression in cuticle and fat body were EF-1α and RPL7A, respectively. For all the samples, RPL13A, EF2, and EF-1α could be considered as the most stable internal reference gene because the minimal STDEV difference was existed among three genes (Figure 3).

### geNorm analysis

**Figure 4:**
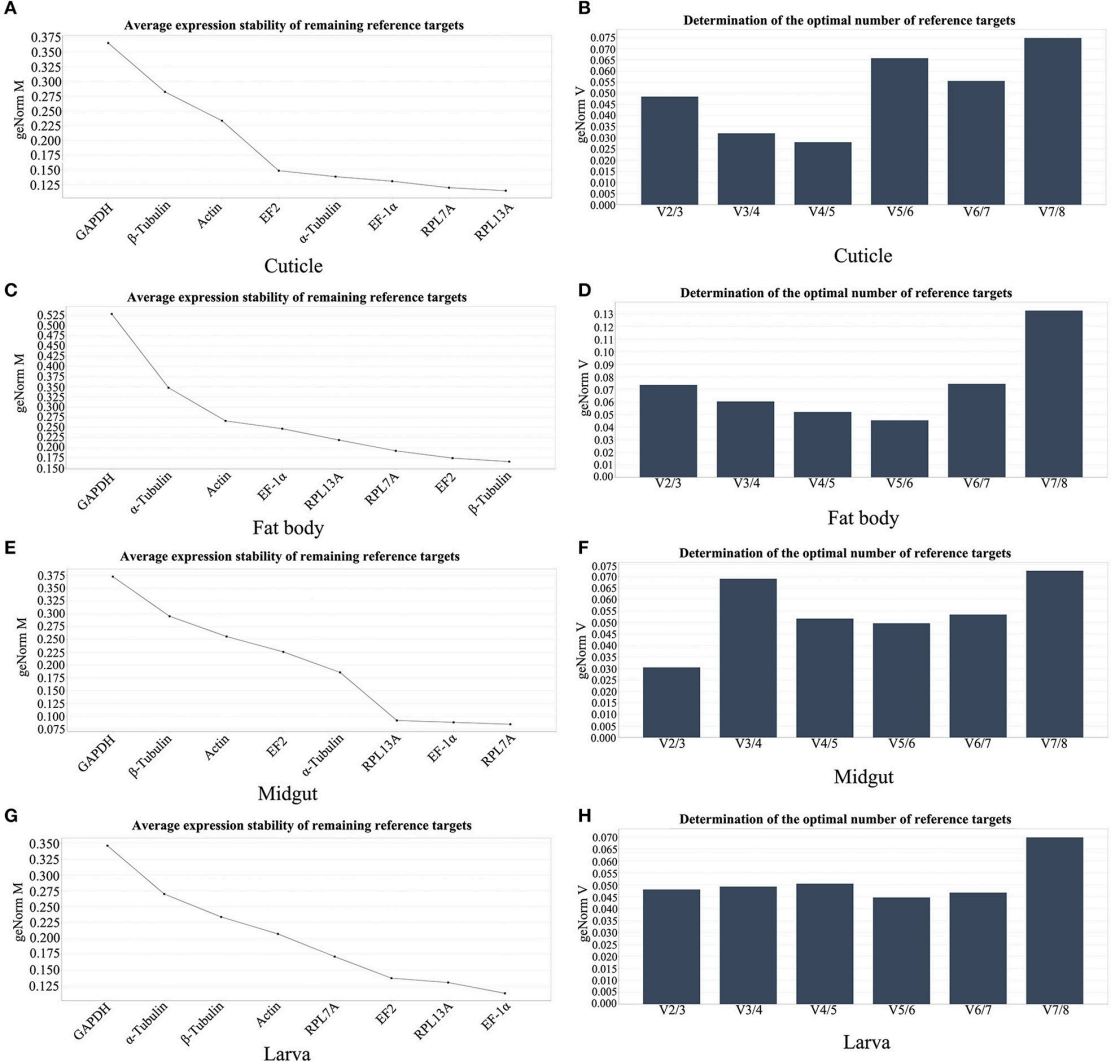
The stability of reference genes expression and the optimal number for normalization analyzed by geNorm software. **(A,C,E,G)** The stability validation of reference genes expression in cuticle, fat body, midgut, and larva. **(B,D,F,H)** pairwise variation values in cuticle, fat body, midgut, and larva. Abbreviations were showed as in Figure 1.

The M value calculated by geNorm software is an important index to evaluate the stability of reference genes and the reference gene with high stability has the lower M value. As shown in Figure 4, RPL7A, β-Tubulin, RPL7A, and EF-1α were evaluated as the most stable reference genes in cuticle, fat body, midgut, and larvae. geNorm also has the function to determine the optimal number of reference genes required for the analysis. As shown in Figures 4B,D,F,G, the pair-wise variation (V_2_/V_3_) in all the tissues and larvae were <0.15, this results revealed that two of the reference genes were required for the reliable normalization of all samples. Therefore, the best combination of reference genes in different samples were: RPL7A and RPL13A for cuticle, β-Tubulin and EF2 for fat body, RPL7A and EF-1α for midgut, EF-1α and RPL13A for larvae.

### RefFinder analysis

**Figure 5:**
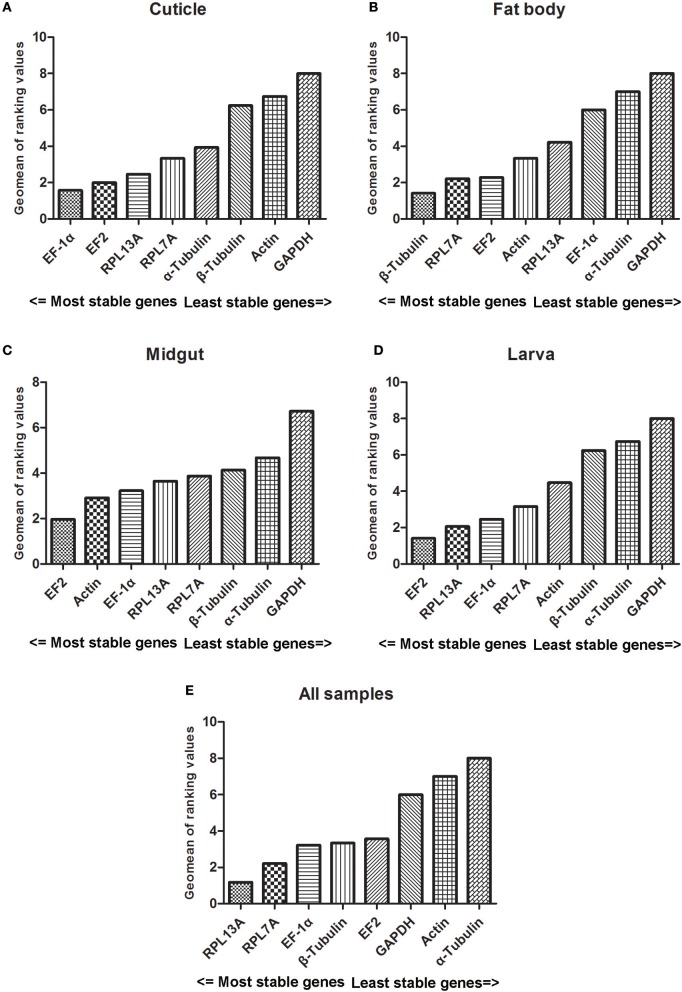
Stability values of the candidate reference genes in different experiment sets generated by RefFinder algorithm. Abbreviations were showed as in Figure 1. Panels **(A–E)** were represented as the stability validation results of reference genes expression in cuticle, fat body, midgut, larva, and all samples.

The stability results of reference genes were also assessed by RefFinder which integrates major computational programs currently available. The comprehensive ranking of reference genes for different tissues were showed in Figure 5. In cuticle samples, EF-1α and EF2 were ranked as the most and second stable reference genes. In fat body samples, β-Tubulin was evaluated as the best reference gene. In midgut and larva samples, EF2 was ranked in the first place. Furthermore, RLP13A was the most stable genes for all the samples and followed with RPL7A and EF-1α.

## Discussion

Compared to some other quantitative methods including Northern blotting, *in situ* hybridization, cDNA arrays, RT-qPCR was more convenience, efficient and widely used. The selection of reference genes could be the critical step to eliminate the variations and get the accurate RT-qPCR results. It was reported that the expression of reference genes is not static in different species, tissues or experimental conditions (Lu et al., 2013), so the stability verifications of reference genes are necessary before evaluating target gene expression levels by RT-qPCR. Recently, more and more research considered this and the stability verifications were also performed in many Lepidoptera insects, for example: *Plutella xylostella* (Fu et al., 2013), *Danaus plexippus* (Pan et al., 2015), *Sesamia inferens* (Lu et al., 2015), *Heliconius numata* (Piron Prunier et al., 2016), *Thitarodes armoricanus* (Liu et al., 2016), *Bombyx mori* (Guo et al., 2016), *Helicoverpa armigera* (Chandra et al., 2017), *Grapholita molesta* (Wang et al., 2017), *Chilo suppressalis* (Xu et al., 2017), *Spodoptera exigua* (Płachetka-Bozek and Augustyniak, 2017), and grassland caterpillars (Zhang et al., 2017). As the important agricultural pest, the identification and validation of reference genes in *S. litura* with three biotic factors and four abiotic treatments were also investigated (Lu et al., 2013).

Azadirachtin is the most efficient and environmentally friendly botanical insecticide for contemporary society which emphasizes green and organic agriculture. It also had significant effects on *S. litura*, including antifeedant, growth inhibitory and so on. Cuticle is the largest organ in insect and azadirachtin has the effect of changing the cuticular protein levels (Yooboon et al., 2015). The midgut was function for food digestion and nutritional absorption and azadirachtin also had many effects on it, including reduced the activities of digestion enzymes and induced apoptosis (Nathan et al., 2005; Shu et al., 2018). In addition, the fat body was also an important organ for metabolic activities and immune response, which speculated it could be involved in the metabolism of azadirachtin (Sun et al., 2017). In this study, these three important tissues or organs were chosen for expression stability validation of eight candidate reference genes after *S. litura* long-time exposure to azadirachtin by NormFinder, BestKeeper, geNorm, Delta CT, and RefFinder. The results indicated that the expression stability obtained by different algorithms were different. Simultaneously, no single internal reference gene showed the most stable expression under different tissues or processing conditions, this was similar to the previous results of *S. litura* (Lu et al., 2013). Therefore, the selection of suitable reference genes should consider all the results of these algorithms.

Ribosomal proteins are abundant in eukaryotic ribosome and function for protein biosynthesis and ribosome assembly, they also play crucial roles in various physiological processes, including cell proliferation and growth (Ladror et al., 2014; Zhou et al., 2015). According to the previous studies, ribosomal proteins were identified as the stable internal reference genes for RT-qPCR. For example, RP49 was considered to be the most stable in *B. mori* and *D. plexippus* for different conditions (Wang et al., 2008; Pan et al., 2015). In addition, RPS18 exhibited the stable expression for most treatments in *Lipaphis erysimi* and *Bactericera cockerelli* (Ibanez and Tamborindeguy, 2016; Koramutla et al., 2016). Other ribosomal proteins were also verified as the stable genes, such as RPL3 for wing discs of *H. numata* (Piron Prunier et al., 2016); RPS13 for *P. xylostella* in different developmental stages (Fu et al., 2013); RPS15 and RPL32 for *H. armigera* in different tissues and treatments (Zhang et al., 2015). In addition, RPL10 was confirmed to be the stable gene in tissues and different populations and RPS3 for the starvation condition of *S. litura* (Lu et al., 2013). Furthermore, RLP13A was determined as the steady in *L. erysimi, T. armoricanus*, and *S. exigua* (Koramutla et al., 2016; Liu et al., 2016; Płachetka-Bozek and Augustyniak, 2017). Simultaneously, RPL7A was also the steady in *S. exigua* and *Lethrus apterus* (Nagy et al., 2017; Płachetka-Bozek and Augustyniak, 2017). In this research, consistent with the above results, we found that RLP13A and RPL7A performed relative higher stable ranking in most samples of *S. litura* under azadirachtin treatments.

Ribosomal translation factors are highly conserved in organisms and response for the protein synthesis by delivering the aminoacyl-tRNAs to ribosome (Mateyak and Kinzy, 2010; Jank et al., 2017). It was reported that elongation factors were suitable as reference genes in different species, tissues, and treatments. Such as, EF1α was recommended for normalization in different tissues, developmental stages, and Bt toxins of *Diabrotica virgifera virgifera* (Rodrigues et al., 2014), the adult tissues of *H. armigera* (Zhang et al., 2015), tissues of *Myzus persicae* (Kang et al., 2016) and most conditions of *D. plexippus* (Pan et al., 2015). Besides, it was evaluated as the normalization gene in different tissues and temperature-stressed samples of *S. litura*. EF2 was also regard as the most stable reference in males of *L. apterus* (Nagy et al., 2017) and all tissue samples of *S. exigua* (Zhu et al., 2014). In our present study, EF1α and EF2 were ranked as the most stable genes in most samples of *S. litura*, which indicated that these two could be used for normalization of RT-qPCR.

In contrast, we found two categories of internal reference genes (skeleton and metabolism-related proteins) which were widely used for RT-qPCR showed instability in most samples, although these genes had been reported as being stable in other species or treatment samples. The skeleton proteins (actin, α-Tubulin and β-Tubulin) could be acknowledged as the target of azadirachtin and the mRNA transcript profiles were affected, which could be the reason for the unstable of these three genes in samples of *S. litura* after azadirachtin treatments (Anuradha and Annadurai, 2008). The metabolism-related protein GAPDH was ranked the least stable genes under azadirachtin, which was consistent with the result of *P. xylostella* for insecticide susceptibility (Fu et al., 2013). The above results indicated that not all housekeeper genes could be used as the reference in a variety of different experimental conditions, and it also highlights the importance for the selection of appropriate reference gene.

In most cases, a single reference gene was used for standardization in RT-qPCR and unsuitable internal reference gene selection could interfere with the accuracy of experimental results. In this study, the software geNorm was used for determining the optimal number of reference genes and we found the index for judging of minimum number V in all the samples were <0.15, which means two reference genes with less expressional variation were need for normalization in *S. litura* under azadirachtin treatments. Therefore, multiple reference genes could be evaluated and used for RT-qPCR to ensure the accuracy of target gene expression.

In summary, eight candidate reference genes of *S. litura* were cloned and identified in this study. In addition, the expression stably of candidate reference genes under azadirachtin treatments were systematically evaluated by five software: geNorm, NormFinder, BestKeeper, Delta CT, and RefFinder. Furthermore, the optimal number of reference genes was also determined. Our results indicated that the best reference genes for RT-qPCR in *S. litura* under azadirachtin treatments could be as followed: EF-1α and EF2 for cuticle samples, β-Tubulin and RPL7A for fat body samples, EF-1α and Actin for midgut, EF2 and RPL13A for lavra and RPL13A and RPL7A for all the samples. Our results not only confirmed the stability of reference genes in *S. litura*, but also provide a basis for the accuracy of target genes and the toxicological mechanism of azadirachtin in insect at the transcriptional level.

## Author contributions

BS and GZ: Conceived and designed the experiments; BS and JZ: Performed the experiments; BS, GC, and XY: Analyzed the data; BS, JZ, RS, and GC: Contributed reagents, materials, and analysis tools; BS: Drafted the manuscript; VS, XY, and GZ: Revised the draft. All authors reviewed the manuscript.

### Conflict of interest statement

The authors declare that the research was conducted in the absence of any commercial or financial relationships that could be construed as a potential conflict of interest.
